# Loss of the Polycomb group protein Rnf2 results in derepression of *tbx*-transcription factors and defects in embryonic and cardiac development

**DOI:** 10.1038/s41598-019-40867-1

**Published:** 2019-03-13

**Authors:** Naomi D. Chrispijn, Dei M. Elurbe, Michaela Mickoleit, Marco Aben, Dennis E.M. de Bakker, Karolina M. Andralojc, Jan Huisken, Jeroen Bakkers, Leonie M. Kamminga

**Affiliations:** 1 Radboud University, Radboud Institute for Molecular Life Sciences, Department of Molecular Biology, Geert Grooteplein 28, 6525 GA Nijmegen, The Netherlands; 2 Radboud University Medical Center, Radboud Institute for Molecular Life Sciences, Geert Grooteplein 28, 6525 GA Nijmegen, The Netherlands; 3 Max Planck Institute of Molecular Cell Biology and Genetics, Pfotenhauerstrasse 108, 01307 Dresden, Germany; 4 Hubrecht Institute, Uppsalalaan 8, 3584 CT Utrecht, The Netherlands; 5 Medical Engineering, Morgridge Institute for Research, 330N Orchard Street, Madison, Wisconsin 53715 USA; 6 Present Address: Department of Human Genetics, Radboud University Medical Center, Nijmegen, The Netherlands; 7 Present Address: Department of Biochemistry, Radboud Institute for Molecular Life Sciences, Nijmegen, The Netherlands

## Abstract

The Polycomb group (PcG) protein family is a well-known group of epigenetic modifiers. We used zebrafish to investigate the role of Rnf2, the enzymatic subunit of PRC1. We found a positive correlation between loss of Rnf2 and upregulation of genes, especially of those whose promoter is normally bound by Rnf2. The heart of *rnf2* mutants shows a tubular shaped morphology and to further understand the underlying mechanism, we studied gene expression of single wildtype and *rnf2* mutant hearts. We detected the most pronounced differences at 3 dpf, including upregulation of heart transcription factors, such as *tbx2a*, *tbx2b*, and *tbx3a*. These *tbx* genes were decorated by broad PcG domains in wildtype whole embryo lysates. Chamber specific genes such as *vmhc*, *myh6*, and *nppa* showed downregulation in *rnf2* mutant hearts. The marker of the working myocard, *nppa*, is negatively regulated by Tbx2 and Tbx3. Based on our findings and literature we postulate that loss of Rnf2-mediated repression results in upregulation and ectopic expression of *tbx2/3*, whose expression is normally restricted to the cardiac conductive system. This could lead to repression of chamber specific gene expression, a misbalance in cardiac cell types, and thereby to cardiac defects observed in *rnf2* mutants.

## Introduction

Proper establishment of cellular identity and subsequent cell type maintenance is crucial during embryonic development and tissue homeostasis. Defects in this complex process can result in disease and/or lethality. Therefore, it is important to study these processes in the context of an *in vivo* system. Modifications of the DNA as well as the associated histones, affect the accessibility of the DNA for the transcriptional machinery. Epigenetic modifiers of the Polycomb group (PcG) protein family are well-known transcriptional silencers, which place specific histone marks^1^. PcG proteins can assemble in two Polycomb Protein Complexes (PRCs): PRC1 and PRC2. PcG proteins were first identified in *Drosophila*, in which mutations of PcG genes resulted in homeotic transformation, by deregulating homeotic (*hox*) genes^2^. In zebrafish the PRC1 core subunits are Rnf2, a Pcgf-family member, a Cbx protein, and a Phc-protein^3^. The core-components of PRC2 are Eed, Suz12, and Ezh1/-2^4^. The canonical view is that PRC2 is first recruited to the chromatin and the enzymatic subunit Ezh2 trimethylates lysine 27 of histone H3 (H3K27me3). PRC1 is recruited to H3K27me3 via its subunit Cbx^5^. The PRC1 subunit Rnf2 mono-ubiquitinylates H2AK119 (H2AK119ub), via its RING-domain and this mark stabilizes H3K27me3^6^. Both H3K27me3 and H2AK119ub are epigenetic marks associated with transcriptional repression^7–10^. H3K27me3 represses gene expression by changing the chromatin structure and by antagonizing the H3K27ac mark, which is a mark known to be present at active enhancers^10,11^. Because H3K27me3 and H3K27ac reside at the same amino acid of the same histone tail, they are mutually exclusive. Furthermore, recent studies showed that the recruitment of PRC1 to the chromatin can also be H3K27me3-independent^12,13^. PRC1 variants that contain subunits which have a DNA-binding domain are described to be involved in this process^14^. H3K27me3-independent recruitment of PRC1 can repress gene expression by different mechanism: by condensation of the chromatin structure, by preventing RNA polymerase II elongation, and by recruiting PRC2^13,15–17^. However, much still remains unknown about the interplay between PRC1 and H3K27me3.

Studies using systems in which PRC1 is disrupted indicate a crucial role in cellular differentiation across species. Mice have two homologous of the RING-domain containing proteins that both can assemble in PRC1: Rnf2 (Ring1b) and Ring1 (Ring1a)^18^. Loss of Rnf2 in mice results in developmental arrest during gastrulation^19^. Murine *Ring1* homozygous mutants are viable^20^, and similar to PcG mutants in *Drosophila*, *Ring1* heterozygous mice display homeotic transformations and skeletal defects^21^. In mice, the loss of Ring1/Rnf2 postnatally results in dental defects, but no lethality, when the mice are studied up to 17 days^22^. Additionally, studies in mouse embryonic stem cells showed that Rnf2 and Ring1 are essential for maintaining cells in a pre-mature state, by repressing genes involved in differentiation pathways^23,24^. In zebrafish, only one Ring1 orthologue is identified, which shows most homology with Rnf2^3^. Therefore, ablation of Rnf2 in zebrafish results in loss of functional PRC1 and the H2AK119ub mark^8^. Zinc-finger nuclease induced *rnf2* null-mutant zebrafish embryos and *rnf2* morphant embryos gastrulate normally, which makes it possible to study development in the absence of Rnf2^8,25^. Rnf2 morphants have an overall normal morphology and, although their primitive erythropoiesis was largely unaffected, the number of hematopoietic stem and thrombocytes was shown to be smaller at 36 hpf^25^. An *rnf2* mutant allele has been generated, and the *rnf2* mutation results in pre-mature stop codon. These *rnf2* null-mutant zebrafish embryos show lethality around 4–5 dpf and display defects in terminal differentiation of the pectoral fins, likely due to interference with Fgf-signaling^8^. In addition, it was found that Cranial Neural Crest (CNC) cells do not properly differentiate into chondrocytes in *rnf2* mutants, resulting in cartilage malformation in the head^26^. These defects in pectoral fin and chondrocyte development upon loss of Rnf2 both arise during terminal tissue differentiation.

To study the role of PRC1 and PRC2 during embryogenesis is challenging due to lethality of mutants in many species before gastrulation^19,27^. Therefore, in this study, *rnf2* mutant zebrafish embryos are used to investigate the effects of loss of Rnf2 on development by studying the transcriptome and correlate this to the Rnf2 binding pattern in wildtype embryos at 3 dpf. We find an important regulatory role for Rnf2 at the chromatin level. The loss of Rnf2 results in upregulation of the genes normally occupied by Rnf2; these include genes associated with transcriptional regulation. In order to gain insight in a tissue specific role of Rnf2 we studied the heart in more detail. Transcriptome analysis of single hearts of wildtype and *rnf2* mutant embryos at 1, 2, and 3 dpf indicates that at 1 and 2 dpf the transcriptional difference between wildtype and *rnf2* mutant hearts are minor and at 3 dpf these differences are more prominent. At 3 dpf upregulation of transcription factors like *tbx2a*, *tbx2b*, and *tbx3a* was detected and, in addition, a downregulation of cardiac chamber genes, such as *nppa* was observed. We suggest that the upregulation of the *tbx* transcription factors is a direct consequence of the loss of Rnf2-mediated repression and we hypothesize that these transcription factors are responsible for the downregulation of chamber genes, resulting in malformation of the *rnf2* mutant hearts. This finding sheds new light on the molecular mechanisms underlying heart development and the role of Rnf2 during vertebrate embryogenesis.

## Results

### Phenotypical differences between *rnf2* mutant and wildtype zebrafish embryos

**Figure 1:**
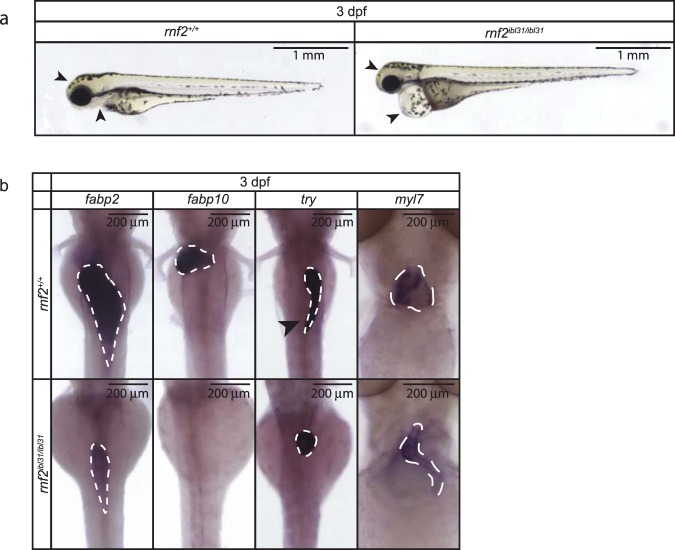
Zygotic *rnf2* mutant zebrafish embryos show a pleiotropic phenotype. (**a)** Lateral view of wildtype embryos (left panel) and *rnf2* mutant embryos (right panel) at 3 dpf. The *rnf2* mutants show a pleiotropic phenotype, including motility problems, craniofacial defects (arrowheads), lack of pectoral fins, and a pronounced heart edema (arrowheads). Not all phenotypes are visible in the pictures. Scale bar = 1 mm. **(b)** Expression of tissue-specific markers was assessed by WISH at 3 dpf in wildtype and *rnf2* mutant embryos. *fabp2*: intestinal marker, *fabp10*: liver marker, *try:* exocrine pancreas marker (arrowhead indicates pancreatic lobe), and *myl7:* cardiomyocyte marker. Scale bar is 200 µm.

To gain additional insight in the role of PRC1 in development we used previously identified *rnf2*^*ibl31/ibl31*^ (referred to as *rnf2*) mutant zebrafish, which harbor a mutation in the enzymatic subunit of PRC1. A 14 base pair deletion in the *rnf2* gene, results in a premature stop codon, and the mutant embryos were shown to lack *rnf2* gene expression at 3 dpf^8^. We observed the same pleiotropic phenotype in *rnf2* mutants, as reported before^8^. This includes motility defects, defects in craniofacial development, the lack of pectoral fins, and a pronounced heart edema (Fig. 1a).

Immunohistochemistry for Rnf2 in wildtype siblings shows that expression of Rnf2 protein at 2 dpf is mainly detected anteriorly and in the notochord (Supplementary Fig. S1, left panel). The *rnf2* mutants lack Rnf2 protein at 2 dpf (Supplementary Fig. S1, right panel).

To obtain more insight in the pleiotropic phenotype we assessed the expression of four organ markers by whole mount *in situ* hybridization (WISH) in wildtype and *rnf2* mutant embryos at 3 dpf. Results from WISH for *fatty acid-binding protein type 2* (*fabp2*), an intestinal marker, suggests a smaller intestine in the *rnf2* mutants compared to the wildtypes (Fig. 1b). Expression of the liver specific marker *fatty acid-binding protein type 10* (*fabp10*) is present in wildtypes, whilst it cannot be detected in *rnf2* mutants. This suggests that liver terminal differentiation is abrogated in *rnf2* mutants. The terminal differentiation marker of the exocrine pancreas, *trypsin (try)*, is present in both wildtype and *rnf2* mutant embryos. However, the shape of the exocrine pancreas is different in *rnf2* mutants: the pancreatic lobe is not detected. Lastly, the expression of the cardiomyocyte marker *myosin light chain* 7 (*myl7*) was assessed. Wildtype embryos show pronounced *myl7* expression in the atrium and the ventricle of the heart. Expression of *myl7* is detected in *rnf2* mutant embryos at 3 dpf; however, the expression pattern of *myl7* in *rnf2* mutants indicates malformation of the heart. The *rnf2* mutant heart shows a stringy morphology and appears smaller based on the *myl7* expression pattern (Fig. 1b).

### Rnf2 binds the same targets as H3K27me3 and H3K27me3 deposition is present in *rnf2* mutants

We next studied the role Rnf2 on the molecular level by identifying its binding on the chromatin, which was not yet assessed in zebrafish. We performed Rnf2 and H3K27me3 chromatin immunoprecipitation followed by deep sequencing (ChIP-seq) at 3 dpf in wildtype and *rnf2* mutant embryos. The canonical pathway describes that the H2AK119ub mark, placed by PRC1 (Rnf2), stabilizes H3K27me3^28^. Additionally, PRC1 variants, containing different subunits than canonical PRC1, have been proposed to be able to recruit PRC2^12,13,29^. Interestingly, numerous studies also reported no effect on H3K27me3 upon loss of PRC1^23,30–34^. Therefore, we studied Rnf2 and H3K27me3 binding patterns and thereby the potential functional redundancy in PRC1 and PRC2 in zebrafish.

**Figure 2:**
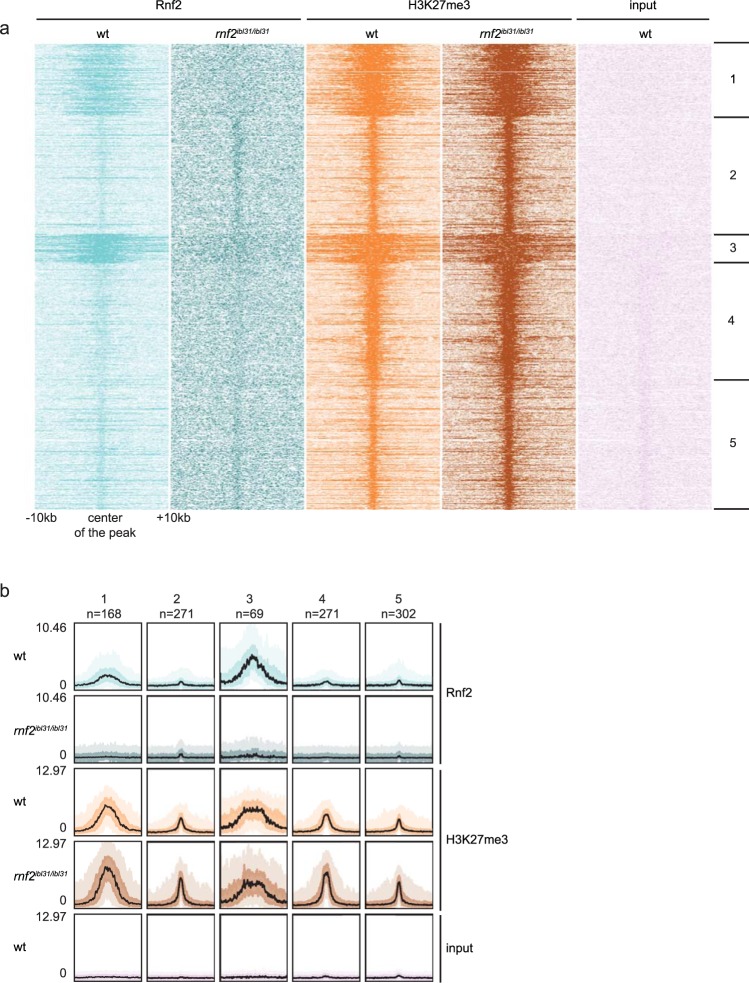
Rnf2 has a similar DNA-binding pattern as H3K27me3 and the presence of H3K27me3 mark is retained upon loss of Rnf2. (**a)** Heatmaps showing *k*-means clustering of Rnf2 and H3K27me3 ChIP-seq peaks in promoter regions in wildtype embryos and *rnf2* mutant embryos at 3 dpf. In the figure 20 kb regions, with a viewpoint around the center of all peaks, are shown. **(b)** Bandplot showing the intensities of the ChIP-seq peaks for Rnf2 and H3K27me3 in wildtypes and *rnf2* mutants at 3 dpf, and the input of the five clusters defined by *k-*means clustering. The number of peak regions included in each cluster is depicted above the plot. The black line indicates the median, the intense color 50% of the peaks, and the light color 90% of the peaks.

To allow quantitative normalization and to demonstrate the efficiency of the method, we added *Drosophila melanogaster* spike-in chromatin during the ChIP-seq procedure^35^. After ChIP-seq, *k*-means clustering revealed five different classes of binding of Rnf2 and H3K27me3 at promoter regions (Fig. 2a). The first cluster represents Rnf2 and H3K72me3 positive promoters. The second, fourth, and fifth cluster contain promoter regions that are positive for H3K27me3 and show close to background levels for Rnf2. The third cluster contains broad PcG domains, in which both Rnf2 and H3K27me3 are present. The intensity of the peaks for Rnf2 and H3K27me3 was analyzed and visualized with bandplots (Fig. 2b). Rnf2 is present at the chromatin in wildtype embryos and is at around background levels in *rnf2* mutants. The levels of H3K27me3 are similar in *rnf2* mutants compared to wildtypes. H3K27me3 presence has been retained upon loss of Rnf2, which could suggest that its deposition does not rely on Rnf2 (Fig. 2b).

### Loss of Rnf2 is associated with upregulation of genes decorated by broad PcG domains

**Figure 3:**
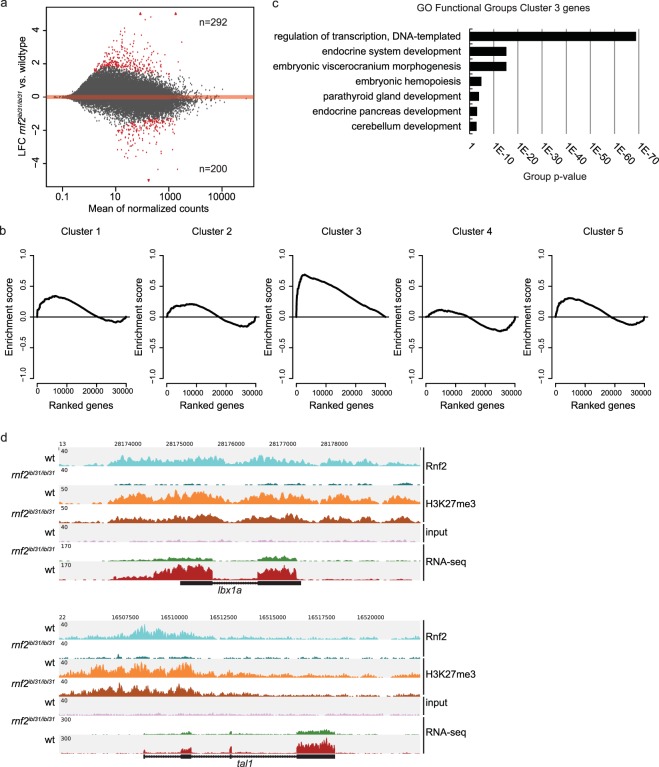
Loss of Rnf2 is directly associated with upregulation of gene expression. (**a**) MA-plot of differentially expressed genes between wildtypes and *rnf2* mutants at 3 dpf. Significantly differentially expressed genes |Log2FC ≥ 1|; padj < 0.1 are highlighted in red. In total 292 genes are upregulated and 200 genes are downregulated. **(b)** Gene Set Enrichment Analysis (GSEA) for the genes whose promoters belong to the five clusters defined in Fig. 2a. Cluster 1, 2, 4, and 5 show a non-significant distribution of the genes based on their expression changes upon the *rnf2* mutation. Genes belonging to the promoters from cluster 3 are enriched for upregulation upon mutation of *rnf2*. p-value = < 0.001; NES = 2.04. **(c)** Gene ontology of biological processes analysis of the 112 genes belonging to cluster 3. **(d)** ChIP-seq and RNA-seq coverage at the *lbx1a* gene and the *tal1* gene in 3 dpf zebrafish embryos. Light blue: Rnf2 ChIP-seq tracks in wildtypes. Teal: Rnf2 ChIP-seq tracks in *rnf2* mutants. Orange: H3K27me3 ChIP-seq track in wildtypes. Brown: H3K27me3 ChIP-seq track in *rnf2* mutants. Lilac: wildtype input. Green: RNA-seq track in wildtypes. Red: RNA-seq track in *rnf2* mutants.

Presence of Rnf2 and H3K27me3 on the chromatin has a repressive effect on the underlying genes^7–10^. Therefore, we compared the transcriptome of wildtype embryos and *rnf2* mutant embryos at 3 dpf. Our data shows both up- and downregulated genes upon loss of Rnf2 (Fig. 3a). In total, 492 genes were found to be differentially expressed (LFC ≥ 1; padj ≤ 0.1). Of these, 292 were identified to be upregulated and 200 genes to be downregulated in *rnf2* mutant embryos (Supplementary Table S1). Hierarchical clustering confirms good homology of the replicates (Euclidian distance; Supplementary Fig. S2a). The organ markers tested by WISH, were also studied in the whole embryo RNA-sequencing dataset. A downregulation of marker expression in *rnf2* mutants was observed for *fabp2*, *fabp10*, and *myl7*. Expression of *try* was not found to be affected by the loss of Rnf2 (Supplementary Fig. S2b).

The promoters of the five different clusters identified after ChIP-seq (Fig. 2a) have been linked to the genes they regulate. The expression of these genes was analyzed by Gene Set Enrichment Analysis (GSEA). We tested the differences in expression between *rnf2* mutants and wildtypes in the five clusters (genes within the five clusters are enlisted in Supplementary Table S2). Genes belonging to the promoters of cluster 1, 2, 4, and 5 do not show significant enrichment for differential regulation upon the *rnf2* mutation (p-value = 0.176, 0.679, 0.605, and 0.249, respectively). Cluster 3 contains 69 promoter regions, regulating 112 genes (‘cluster 3 genes’). This cluster is significantly enriched with genes that are upregulated in the *rnf2* mutant embryos at 3 dpf (p-value < 0.001) (Fig. 3b). Gene ontology analysis shows that these ‘cluster 3 genes’ are associated with regulation of transcription and embryonic organ development (Fig. 3c). Examples of tracks of two ‘cluster 3 genes’ are shown in Fig. 3d. These genes are decorated by Rnf2 and H3K27me3 and the RNA-seq tracks indicate a significant upregulation of gene expression in *rnf2* mutants at 3 dpf. In total, 24 genes were identified in cluster 3 that were significantly upregulated in *rnf2* mutant embryos which are decorated by Rnf2 in the promoter region of wildtype embryos at 3 dpf (LFC ≥ 1; padj ≤ 0.1) (Supplementary Table S3).

### The cardiac phenotype in *rnf2* mutant zebrafish embryos shows looping defects

To gain insight into a tissue specific role of Rnf2 during development, we performed more extensive studies on the heart, which was one of the organs severely affected upon the *rnf2* mutation. The heart is a well-studied organ in zebrafish, due to its similarity in cardiac development to other species and its regenerative capacities. Since we observe a severe cardiac phenotype upon loss of Rnf2, we studied a potential role of Rnf2-mediated regulation of heart development.

**Figure 4:**
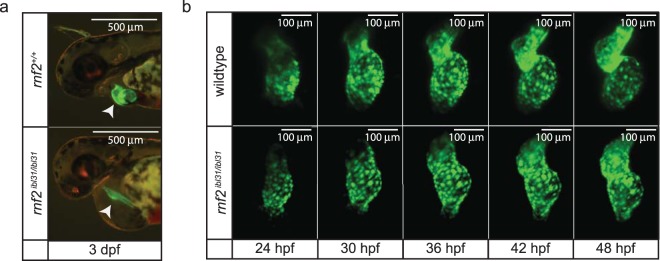
Zebrafish *rnf2* mutant embryos show cardiac looping defects. (**a**) Fluorescent images of wildtype and *rnf2* mutant sibling embryos in a *Tg(myl7::GFP)* background at 3 dpf. Scale bar is 500 µm. **(b)** Stills of live-imaging by light sheet microscopy of wildtype and *rnf2* mutant hearts in a *Tg(myl7::GFP)* background starting at 1 dpf. Scale bar is 100 µm.

We analyzed the development of the heart of wildtype and *rnf2* mutant embryos at 3 dpf in a *Tg(myl7::GFP)* background. This transgene specifically marks cardiomyocytes^36^. During normal cardiac development, cardiac looping ensures the proper positioning of the atrium and ventricle and it marks the transition from a linear heart tube to a two-chambered heart separated by the atrioventricular canal (AVC), which occurs between 28 and 50 hours post fertilization^37^. In contrast to the wildtype situation, the hearts of *rnf2* mutant embryos display defective looping morphogenesis, resulting in a stringy heart phenotype with no clear defined cardiac chambers or AVC at 3 dpf (Fig. 4a). To investigate the underlying developmental dynamics, we used high speed selective plane illumination microscopy (SPIM) to time-lapse image wildtype and *rnf2* mutant embryos from 1 to 2 dpf (Fig. 4b). Indeed, whereas wildtype embryos showed cardiac looping around 36 hpf the mutants fail to loop properly (Fig. 4b).

### Single HeartsRNA-seq highlights differences between wildtype and *rnf2* mutant hearts over time

We next aimed at getting a better understanding of the molecular mechanisms underlying the heart defects detected in *rnf2* mutants. To start with, we show that disturbing the epigenetic repressor Rnf2 shows a global effect on gene expression, especially of genes decorated by Rnf2 in wildtype embryos (Fig. 3a). In order to address this finding and in connection with the observed cardiac phenotype, we took genes positive for Rnf2 in their promoter, as identified by ChIP-seq (n = 206), and all genes detected by RNA-seq (n = 32,266) and searched for the presence of heart transcription factors (n = 18) within these two groups^38^, finding a significant enrichment of this category (chi-squared test; p-value < 0.001).

**Figure 5:**
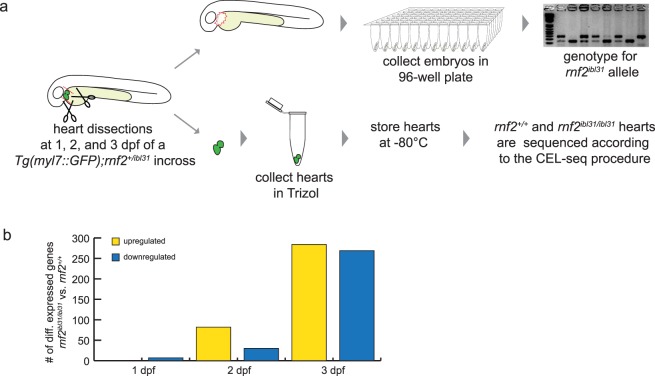
Single HeartsRNA-seq is used to assess transcriptional differences between wildtype and *rnf2* mutant hearts over time. (**a**) Workflow of Single HeartsRNA-seq. Zebrafish hearts were manually dissected at 1, 2, and 3 dpf as described previously^85^. The remaining tissue was used for genotyping and the *rnf2* mutant and wildtype hearts were sequenced. **(b)** Differentially expressed genes |Log2FC > 0|; padj < 0.01 between *rnf2* mutant and wildtype hearts are visualized in the bar graphs. Yellow: upregulated (up) in *rnf2* mutants compared to wildtypes. Blue: downregulated (dn) in *rnf2* mutants compared to wildtypes.

To gain more detailed insight into the role of Rnf2 in cardiac development, single embryonic hearts, both from *rnf2* mutant and wildtype, serve as a useful model. The transcriptome of single hearts dissected from wildtype and *rnf2* mutants was assessed, using a low-input RNA-seq method based on CEL-seq^39–42^. This method is very suitable for the number of cells present in a single embryonic heart, which is between 150 and 350 cells at 1 to 3 dpf^41,43,44^. We performed manual dissection of single embryonic hearts at 1, 2, and 3 dpf and prepared the individual *rnf2* mutant and wildtypes dissected hearts for RNA-sequencing (Single HeartsRNA-seq, Fig. 5a). In total 63 single hearts were sequenced (Supplementary Fig. S3a). Samples were filtered based on the number of mRNAs they express, and genes were filtered based on the number of samples that express them (see Materials and Methods and Supplementary Fig. S3b). In total 5 samples were excluded and at least 8 replicates remained per genotype per developmental time point (Supplementary Fig. S3c). After this, gene counts were normalized to avoid, among others, differences derived from unequal amounts of cardiac tissue developed by the *rnf2* mutant and wildtype embryos. Hierarchical clustering based on Euclidian Distances at the 3 different developmental time points indicates that differences between wildtype and *rnf2* mutant hearts are minor at 1 and 2 dpf and more pronounced at 3 dpf (Supplementary Fig. S3d–f). This is also reflected in the number of genes that are differentially expressed at these different time points (Fig. 5b). At 1 dpf no genes were found to be upregulated and 7 genes were significantly downregulated (|Log2FC > 0|; padj < 0.01). This number increased to 82 upregulated and 30 downregulated genes at 2 dpf. At 3 dpf 284 genes were detected to be significantly upregulated and 269 genes to be significantly downregulated in *rnf2* mutant heart. Overall, we observed that the number of differentially expressed genes between wildtype and *rnf2* mutant hearts increased over time (Fig. 5b, Supplementary Table S4).

### Cardiac chamber identity is disrupted in *rnf2* mutants at 3 dpf

**Figure 6:**
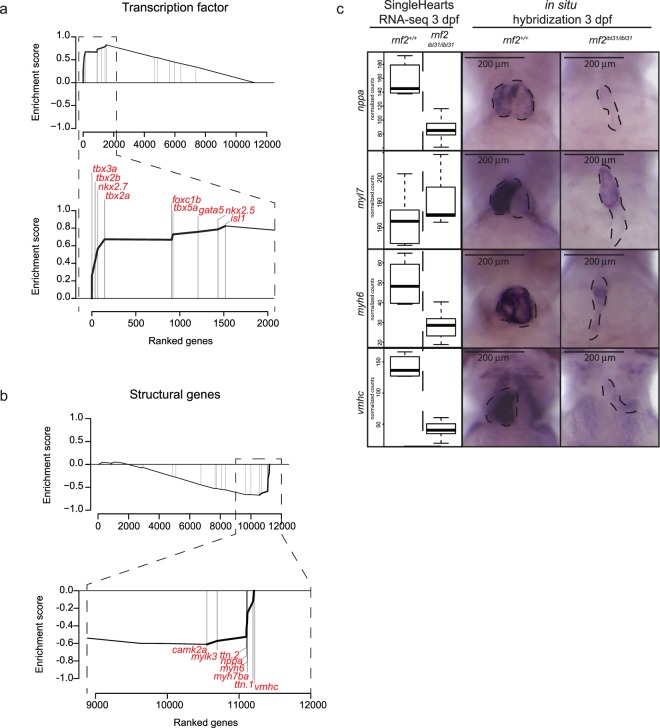
Cardiac chamber identity is disrupted in *rnf2* mutants at 3 dpf. (**a**) GSEA using the list of cardiac genes reported by Hill *et al*.^38^ indicates that the group of transcription factors is significantly enriched in being upregulated in *rnf2* mutant hearts at 3 dpf as detected by Single HeartsRNA-seq (FDR q-value = 0.001; NES = 1.96). The leading transcription factors (n = 9) are listed in the zoom of the GSEA plot. **(b)** GSEA indicates the annotation ‘Structural Group’ genes (n = 23) to be enriched for downregulation upon the *rnf2* mutation in hearts at 3 dpf. The leading genes (n = 8) are listed in the zoom of the GSEA plot. FDR q-value = 0.054; NES = −1.67. **(c)** Normalized counts as found by Single HeartsRNA*-*seq for *nppa*, *myl7*, *myh6*, and *vmhc* at 3 dpf in wildtype and *rnf2* mutant hearts with their accompanying whole mount *in situ* hybridizations. Scale bar is 200 µm.

Recently, Hill *et al*. generated a hand-curated list of cardiac markers^38^. This list contains genes that are expressed in the developing heart and includes 26 annotations about the function and the location of these genes. These 26 annotations were studied for the gene expression changes of the genes falling into these categories. We used GSEA using the Single HeartsRNA-sequencing results of 3 dpf hearts (Fig. 6a). This analysis revealed that *rnf2* mutant hearts are enriched for genes expressed in the atrioventricular canal (FDR q-value = 0.089; NES = 1.46) and for heart transcription factors (FDR q-value = 0.001; NES = 1.96; Fig. 6a). These two annotations show overlap in the genes they contain. Three transcription factors that repress myocardial genes (*tbx2a*, *tbx2b*, and *tbx3a*) are present in the top 4 of genes that are upregulated in *rnf2* mutant hearts. Single HeartsRNA-seq results at 1, 2, and 3 dpf indicate that the expression of *tbx3a* is significantly upregulated at 2 dpf and that expression of *tbx2a*, *tbx2b*, and *tbx3a* is significantly upregulated at 3 dpf in *rnf2* mutants (|Log2FC > 0|; padj < 0.01, Supplementary Fig. S4).

The GSEA using the 3 dpf Single HeartsRNA-sequencing results additionally indicated an enrichment for downregulation of structural genes (FDR q-value = 0.054; NES = −1.67; Fig. 6b) and myocardial genes (FDR q-value = 0.043; NES −1.62). The group of genes downregulated upon the *rnf2* mutation is enriched in structural genes such as: *vmhc*, *ttn*.*1*, *myh7ba*, *myh6*, *nppa*, *ttn*.*2*, *mylk3*, and *camk2a* (Fig. 6b). Amongst the genes that are leading there is the marker for the working myocard *nppa*, the atrium marker *myh6*, and the ventricle marker *vmhc*. We analyzed the expression of *nppa*, *myl7*, *myh6*, and *vmhc* by Single HeartsRNA-seq and additionally tested the spatio-temporal expression of these genes by whole mount *in situ* hybridization (WISH) (Fig. 6c, Supplementary Fig. S5). At 1 and 2 dpf the expression differences between wildtype and *rnf2* mutant hearts for these four genes are relatively small. We found *nppa* and *vmhc* to be significantly downregulated in *rnf2* mutant hearts at 1 dpf and 2 dpf, respectively. Single HeartsRNA-seq results at 3 dpf indicate that the expression of *nppa*, *myh6*, and *vmhc* is significantly decreased in *rnf2* mutants (|Log2FC > 0|; padj < 0.01, Fig. 6c).

## Discussion

### *Rnf2* mutants show a pleiotropic phenotype

In this study zygotic *rnf2* mutant embryos (*rnf2*^*ibl31/ibl31*^) are used as a model for loss of PRC1 and H2AK119ub^8,26^. These mutant embryos display defects in the maintenance of cellular identity and organ integrity^8,26^. Rnf2 is the only catalytic subunit of PRC1 in zebrafish and therefore disrupting Rnf2 is informative of PRC1’s putative role in zebrafish development.

The *rnf2* mutation results in a pleiotropic phenotype in zebrafish embryos at 3 dpf^8^. A multitude of genes and processes were described to be affected upon the loss of Rnf2^8,26^, however no genome-wide molecular expression datasets are available for these mutants. Since Rnf2 is an epigenetic modifier, ChIP-sequencing can give insight in its mode of action and RNA-sequencing can elucidate its downstream effects. With bright field microscopy and WISH analyses we studied the *rnf2* mutant phenotype on a global level. Next to the described phenotypes of *rnf2* mutants^8,26^, we observed a heart edema accompanied by a tubular heart. WISH analyses suggest developmental organ defects of the intestine, pancreas, and liver at 3 dpf. If the observed differences are due to organ defects, they could result from defects in differentiation, cell proliferation, or tissue maintenance, as well as a combination of these three, as PcG proteins are described to play a role in these processes^45^. In this study we focused on heart development.

### H3K27me3 is retained upon *rnf2* mutation

H2AK119ub, the mark deposited by PRC1, and more specifically, by its enzymatic subunit Rnf2, is described to stabilize H3K27me3^9^. In addition, also a role for PRC1 in the recruitment of PRC2 is proposed^12,13^. Therefore, loss of H3K27me3 was considered to occur in the absence of Rnf2. However, many studies also reported that the loss of PRC1 does not affect H3K27me3 deposition^23,30–34^. We used our PRC1 null model (*rnf2* mutants) to study this process *in vivo* and show that H3K27me3 deposition at 3 dpf is retained upon loss of Rnf2/PRC1, which is thus in line with the majority of these previous reports^23,30–34^. Based on our data one could even argue that the *rnf2* mutation results in a slight increase in H3K27me3 levels. We study whole embryo lysates and therefore this slight increase can be due to more H3K27me3 deposition within the cell, however it can also be due to changes in the abundance of cells that repress these genes via an H3K27me3-mediated manner. This could also be the reason why we do not detect a loss of H3K27me3 in the absence of Rnf2, as changes in individual cells will be overshadowed by the signal detected in the bulk of all cells analyzed.

### The *rnf2* mutation results in derepression of genes decorated by Rnf2

A subset of genes was found to lose Rnf2-mediated repression and this subset was detected to be significantly upregulated in *rnf2* mutants; however, they retain H3K27me3-mediated repression at the whole embryo lysate level. A potential explanation for gene upregulation, whilst they are decorated by H3K27me3, could be derived from the type of sample that has been used. The samples are lysates from whole embryos at 3 dpf, in which many cell types are present and the sequencing results give an average of the signal coming from across all those different cell types. Therefore, overall gene upregulation could be caused by cells in which these genes are not repressed by H3K27me3.

### The role of epigenetics in heart development

The role of epigenetics in cardiac development has gained more attention over the years, as reviewed by Vallaster *et al*., and Shirai *et al*.^46,47^. Zebrafish hearts are of high interest due to their regenerative capacity and epigenetics is also implied to be important in this process, as reviewed Quaife-Ryan *et al*.^48^. Therefore, it is of essence to better understand the role of epigenetics in heart tissue specification, maintenance, and regeneration. To unravel important regulators of cardiac development, studies have been performed that aimed to make a roadmap of the transcriptome and epigenome during myocardial differentiation^49,50^. Other approaches focus on chromatin remodelers and the identification of enhancers to gain insight in the role of epigenetics in heart development^51,52^. Many histone modifiers are described to play a role during cardiac development, such as histone deacetylases, HDACs^53,54^, H3K4me3 methyltransferase^55^, and also the PRC2-component Ezh2^56–58^.

The PRC1-variant containing Mel18 was described to be essential for specification of mesodermal cell fate, by preventing alternative lineage commitment^29^. In line with that, the PRC1-component Bmi1 was shown to act as a barrier during cardiac reprogramming in mouse cells^59^. Interestingly, a role in cardiac development *in vivo* for both Mel18 and Bmi1 has not been established^60,61^. That Mel18-PRC1 and Bmi1-PRC1 do not play a role in cardiac development does not exclude the possibility that any of the other PRC1 variants do. A role for Rnf2 in cardiac development has not been described *in vivo*, so far. In our current study we show that depletion of Rnf2 affects heart morphology and gene expression during zebrafish development.

A study on H3K4me3 methyltransferases in zebrafish heart development has shown that a decrease in H3K4me3 results in a linear shaped heart, similar to the *rnf2* mutants. However, in that study the heart markers *myl7*, *vmhc*, and *myh6* were reported not to be affected at 2 dpf upon decrease of H3K4me3^55^. This is in contrast to the *rnf2* mutants, as we observe at 2 dpf significant downregulation of *vmhc* expression. A previous study from our lab using maternal zygotic *ezh2* mutants also shows a linear shaped heart upon the mutation of the catalytic subunit of PRC2. Altogether, these results indicate that a similar phenotype can have different causes and different epigenetic signatures, as both a loss of H3K4me3, H3K27me3, and H2AK119ub result in a linear shaped heart phenotype^55,58^.

### Repression of *tbx*-genes by Rnf2 is important for cardiac development

At early stages (1 and 2 dpf) we found the expression of cardiac genes in *rnf2* mutant hearts to be more or less similar to wildtype hearts, therefore we hypothesize that maternal *rnf2* RNA and Rnf2 protein are sufficient for correct cell specification in the *rnf2* mutants. On top of that, as most PcG proteins, Rnf2 is expected to be mostly involved in tissues maintenance rather than tissue specification^8,19,23,26,58^.

Since we observe an upregulation of *tbx*-genes in a system in which we mutate a transcriptional repressor, we analyzed our Rnf2 ChIP-seq results on whole embryo lysates and found that the *tbx3a* gene is bound by Rnf2 and decorated by H3K27me3 in the wildtype situation. This observation strongly hints towards Polycomb-mediated regulation of Tbx3. Literature describes that this is not zebrafish-specific, since Pcl2 (PRC2 subunit) knock-out murine ESC show an upregulation of *Tbx3*^62^. Research has revealed that when Tbx2/3 forms a complex with Gata and Nkx, it locally represses chamber myocardial gene expression and thereby enhances the formation of the conduction system^63^. Tbx2/3 are described to directly repress, amongst others, the myocardial gene *nppa* to allow for the formation of the conductive system in the heart^64,65^. *Z*ebrafish *rnf2* mutants show a malformed heart and our 3 dpf Single HeartsRNA-seq dataset shows that *nppa* is significantly downregulated, which we validated by WISH experiments. Tbx2/3 overexpression in mouse embryos results in a heart looping defect and the lack of cardiac chambers^66,67^. In these murine embryos the chamber-myocardial gene program is not correctly set up. Interestingly, the *rnf2* mutant zebrafish embryos also show a heart in which the atrium and ventricle are not well developed.

Furthermore, Tbx5 competes with Tbx2/3 to form a complex with Nkx and Gata, and this complex has an activating effect on gene expression^66^. Early stages of *rnf2* mutants show correct *tbx5* levels, most likely due to the maternal load of *rnf2*^8^. However, at 3 dpf we detect *tbx5a* to be upregulated in the heart upon the *rnf2* mutation and whole embryo lysate ChIP-seq indicates that the *tbx5a* gene is decorated by Rnf2 and H3K27me3. Overexpression of *tbx5 in vitro* has been reported to represses proliferation and cell growth^68^, which is in line with our observations. In contrast, *rnf2* mutant zebrafish were reported to have greatly reduced expression of *tbx5* at the pectoral fin mesenchyme, which is correlated to the absence of pectoral fins in these mutants^8^ and interestingly, the loss of Tbx5 results in a stringy heart in zebrafish embryos^69^.

These studies and our observations indicate that tight regulation of *tbx2a*, *tbx2b*, *tbx3a*, and *tbx5a* is required for proper heart development. Since three Tbx2/3 variants are overexpressed in the *rnf2* mutant heart to a larger extent than *tbx5a*, we suggest that overexpression of *tbx2/3* is the main driver of the observed myocardial phenotype. We therefore postulate that the overall downregulation of myocardial genes is the result of inadequate activation or maintenance of the chamber-myocardial gene expression program, which results in defects in maintenance of cell identity. Studies by others indicate that Rnf2 is an important player in the maintenance of tissue integrity in a wide variety of systems, and the zebrafish data on single hearts from *rnf2* mutants adds to this list^8,19,23,26^. We hypothesize that the molecular pathway that allows for the formation of the conductive system is partially regulated by Rnf2 and that this ensures the correct balance in chamber and conductive cell identity within the heart. Disruption in this balance results in defects in cardiac development and functioning.

## Methods

### Zebrafish genetics and strains

Zebrafish (*Danio rerio*), were housed at 27.5 °C in a 14/10 h light/dark cycle. The evening before spawning, one male and one female were placed into a tank with a divider and the following morning, at the moment the light switched on, the fish were placed together for breeding. Embryos were collected and staged according to Kimmel *et al*.^70^. The *rnf2*^*ibl31/ibl31*^ zebrafish were out-crossed with wildtype (TLF) or with *Tg(myl7::GFP)*^8,36^. All methods were carried out in accordance with relevant guidelines and regulations of national animal welfare laws.

### Genotyping

DNA was purified from embryos or from caudal fin tissue, taken from anesthetized adult zebrafish. Genotype analysis was performed by PCR using the primer set forward: 5′-TCTAAGCGCTCTCTTCGTCCAGA-3′ and reverse: 5′-ACAAGAGGATTTGTAACAAAGCCG-3′, followed by digestion of the PCR product with restriction enzyme *Taq1* to identify the *rnf2*^*ibl31/ibl31*^ allele^8^. The agarose gel was imaged using the Gel Doc XR+ Imaging System (Bio-Rad), in combination with Image Lab Software (Bio-Rad). After acquisition, the image color was inverted and the levels were adjusted, using Photoshop, to visualize all bands for correct genotyping.

### Whole mount *in situ* hybridization

Dechorionated embryos were fixed overnight at 4 °C in 4% PFA (Aurion, 151710) in PBST (PBS with 0.1% Tween-20), after which they were gradually transferred to and stored in 100% methanol. To prevent probe trapping, the heart edema of 3 dpf *rnf2* mutant embryos was pierced with watchmaker forceps (INOX5). Embryos were treated with proteinase K. Whole mount *in situ* hybridization was performed as described previously^71^. *Trypsin* and *fabp10* probes were generated by PCR from cDNA from 1 dpf wildtype embryos using the following primers: forward *trypsin* CAGG CCCTTTAGTGAGGGTTAATT TGTCTGCTGCTCACTGGTAC; reverse *trypsin* CAGG TAATACGACTCACTATAGGG GTCCTTGCCTCCCTCCATAA. Forward *fabp10* CAGG CCCTTTAGTGAGGGTTAATT GTTGAGCTTCTCCAGAAAGCATG, reverse *fabp10* CAGG TAATACGACTCACTATAGGG GATCATGGTGGTTCCTCCGA. T7 polymerase was used to generate the anti-sense probes. After WISH the embryos were mounted in 4% methylcellulose and imaged by light microscopy on a Leica MZFLIII, equipped with a DFC450 camera. The embryos were genotyped after imaging.

### Immunostainings

Dechorionated embryos were fixed overnight in 4% PFA in PBST at 4 °C. After fixation, embryos were gradually transferred to and stored in 100% methanol. Before immunostaining embryos were transferred stepwise to PBST. Rabbit anti-Ring1b antibody from Cell Signaling Technology was used (RING1B Cell Signaling D22F2 1:4000). Antibody incubation was followed by a secondary antibody and subsequent DAB staining (EnVision+ System-HRP (DAB) k4010). The embryos were mounted in 4% methylcellulose and imaged by light microscopy on a Leica MZFLIII, equipped with a DFC450 camera. The embryos were genotyped after imaging.

### ChIP-sequencing

Embryos from a *rnf2* heterozygous incross were sorted for the *rnf2* pleiotropic phenotype at 3 dpf. These phenotypical mutants are used for the *rnf2* mutant sample. The *rnf2* mutation has a 100% penetrance and no false positives have been detected by genotyping after phenotypic screening. As controls we used a wildtype strain from the same genetic background. Pools of 80 to 100 embryos of 3 dpf were fixed, deyolked, and homogenized using pestles and sonicated to release and isolate the chromatin. Chromatin was stored at −80 °C. For spike-in experiments 30 µg zebrafish chromatin was mixed with *Drosophila* spike-in chromatin and incubated overnight with H2Aγ and RING1B antibodies (H2Aγ Active Motif 104597; RING1B cell signaling D22F2) or with H2Aγ and H3K27me3 antibodies (H2Aγ Active Motif 104597; Millipore 07–449). After overnight antibody incubation, the chromatin was extensively washed. The resulting ChIP-DNA was used as input for KAPA-HYPERprep library preparation. Libraries were paired-end sequenced (43 bp read-length) on an Illumina NextSeq500 platform. For wildtype and *rnf2* mutant samples, two and three replicates were used, respectively.

### ChIP-sequencing analyses

In order to avoid biases due to differences in the efficiency of the sequencing runs, spike-in ChIP-seq reads were mapped to the *Drosophila melanogaster* genome v6 using bwa mem version 0.7.15^72^ with default settings. Multimapping reads were excluded using samtools version 1.3.1^73^ and duplicated reads were removed with Picard (http://broadinstitute.github.io/picard/). Once we obtained the number of reads mapped per sample, we normalized them based on the sample of each experiment with lowest number of mapped reads, by removing random reads accordingly. After this, remaining ChIP-seq reads were mapped to the GRCz10/danRer10 using bwa mem version 0.7.15^72^ with default settings. Multimapping reads were excluded using samtools version 1.3.1^73^ and duplicated reads were removed with Picard (http://broadinstitute.github.io/picard/). Peaks were called using MACS 2.1.1.20160309^74^ relative to the input track using the options -f BAMPE -g 1.3e9 -q 1e-2–broad –broad-cutoff 1e-1. Peaks 1 kb or closer from each other were merged, and H3K27me3 peaks narrower than 100 nt were discarded. Intersecting peaks were considered for replicates using GenomicRanges^75^. Peaks overlapping peaks called in the input track were excluded. Clustering of peaks was done using the union of the remaining peaks found in wildtype Rnf2 and H3K27me3 ChIPs and *rnf2* mutant H3K27me3 ChIP, considering only those overlapping promoter regions (400 nt upstream–100 nt downstream of the transcription start site). Clustering and visualization of the peaks (i.e. heatmaps, bandplots and profiles) was done using fluff version 2.1.3^76^. GO term analysis for biological process on genes of cluster 3 was performed with Cytoscape 3.3.0 using the ClueGO 2.2.4 plugin with default settings. To reduce redundancy in biological process GO terms, GO term fusion and GO term grouping was applied and the groups were plotted with group p-value corrected with Bonferroni step down.

### Gene set enrichment analyses

For whole embryo lysates, gene set enrichment analyses were performed using the GSEA software version 3.0 from the Broad Institute^77^ using default parameters, comparing gene counts from *rnf2* mutants and wildtypes, normalized with DESeq2 1.28.0^78^. As gene sets, genes belonging to the promoter regions of the five different clusters were considered. For single hearts, gene counts from 3 dpf wildtype and *rnf2* mutant samples were compared after data normalization with Monocle version 2.4.0^79^. As gene sets, a hand-curated list of heart markers^38^ classified by “Annotation” was used.

### RNA-sequencing of whole embryo lysates

Embryos from a *rnf2* heterozygous incross were sorted for the *rnf2* phenotype at 3 dpf. These phenotypical mutants are used for the *rnf2* mutant sample. As controls a wildtype strain from the same genetic background was used. Pools of 11 to 23 embryos of 3 dpf were homogenized in TRIzol. The ZYMO RNA microprep kit was used to isolate RNA and treat the samples with DNAseI. Subsequently, 750 ng RNA was used as starting material. rRNA was depleted using the Illumina RiboZero kit, followed by fragmentation, cDNA synthesis, and KAPA-HYPERprep library preparation. Libraries were paired-end sequenced (43 bp read-length) on an Illumina NextSeq500 platform. For wildtype and *rnf2* mutant samples, eight and seven replicates were used, respectively.

### RNA-sequencing analyses

RNA-seq reads were mapped to the *D*. *rerio* genome (GRCz10/danRer10) with the Ensembl gene annotation v87 using STAR^80^ version 2.5.2b with default parameters and –quantMode on “GeneCounts” to obtain quantification of expression levels. Analysis of differentially expressed genes was done with DESeq2 1.28.0^78^ after removing the batch effect on all samples with RUVSeq 1.10.0^81^.

### Fluorescent imaging

Embryos from a *Tg(myl7::GFP);rnf2* heterozygous incross were anaesthetized in MS-222 and embedded in 1.5% low-melting-point agarose (Sigma). The embryos were imaged by light microscopy on a Leica MZFLIII, equipped with a DFC450 camera. The embryos were genotyped after imaging.

### SPIM-imaging

Embryos from a *Tg(myl7::GFP)*_*rnf2* heterozygous incross were injected with α-Bungarotoxin at the one-cell stage^82^. As controls we used embryos from a *Tg(myl7::GFP)* incross, which were also injected with α-Bungarotoxin at the one-cell stage^82^. At 1 dpf the embryos were embedded for SPIM imaging in 1.5% low-melting-point agarose (Sigma) in FEP tubes (Bola, S1815-04). We used the custom build multidirectional selective plane illumination microscopy (mSPIM) as described before^83^. Photos were taken with a 20-minute interval and the images were synchronized. The stages in which both the ventricle and atrium are dilated were used for data visualization by Imaris software (bitmap). The embryos were genotyped after imaging.

### Single HeartsRNA-sequencing

Hearts were manually dissected from 1, 2, or 3 dpf *Tg(myl7::GFP)* positive embryos from an *rnf2* heterozygous incross using watchmaker forceps (INOX5) and placed into Eppendorf LoBind tubes with TRIzol (Ambion), rapidly frozen in liquid nitrogen, and stored at −80 °C prior to further processing. The remainder of the embryos was individually collected in methanol and used for genotyping. RNA was extracted from the wildtype and *rnf2* mutant hearts using TRIzol reagent (Ambion) according to the manufacturer’s manual. After RNA extraction, pellets were resuspended with barcoded primers. Primers consisted of a 24 bp polyT stretch, a 4 bp random barcode, a unique 8 bp sample-specific barcode, the 50 Illumina adaptor (as used in the TruSeq small RNA kit), and a T7 promoter for *in vitro* transcription^84^. The RNA samples were subsequently reverse transcribed, pooled, and *in vitro* transcribed for linear amplification with the MessageAmpII kit (Ambion) according to the CEL-seq protocol^42^. Illumina sequencing libraries were prepared with the TruSeq small RNA sample prep kit (Illumina) and sequenced single-end at 75 bp read length on an Illumina NextSeq. 500 instrument.

### Single HeartsRNA-sequencing analyses

Raw reads were processed to obtain expression levels following the pipeline designed for the CEL-Seq method^42^ using the scripts available at https://github.com/yanailab/CEL-Seq-pipeline. Data normalization and differential gene expression analyses were done with Monocle version 2.4.0^79^. Samples were filtered according to +/−2 SD cut-off in the amounts of mRNA that is detected in the sample after log transformation. Genes expressed in at least 25% of the samples at a given developmental stage were used for differential gene expression analysis and GSEA.

## Supplementary information

Supplementary Information

Supplementary Dataset 1

Supplementary Dataset 2

Supplementary Dataset 3

Supplementary Dataset 4

## Data Availability

The ChIP-seq data and RNA-seq data from whole embryo lysates and the single hearts discussed in this manuscript have been deposited in NCBI’s Gene Expression Omnibus and are accessible through GEO Series accession number GSE114038.
