# Supplementary material for: Loss of the Polycomb group protein Rnf2 results in derepression of tbx-transcription factors and defects in embryonic and cardiac development
Source: Sci Rep. 2019 Mar 13;9:4327. doi: 10.1038/s41598-019-40867-1 (PMC6416260; doi:10.1038/s41598-019-40867-1)
Supplement: Supplementary file 1 — Supplementary Information [file 41598_2019_40867_MOESM1_ESM.pdf]

Supplemental Information

Loss of the Polycomb group protein Rnf2 results in derepression of *tbx*-transcription factors and defects in embryonic and cardiac development

Naomi D. Chrispijn, Dei M. Elurbe, Michaela Mickoleit, Marco Aben, Dennis de Bakker, Karolina M. Andralojc, Jan Huisken, Jeroen Bakkers, Leonie M. Kamminga

Supplementary figure legends

Supplementary Figure S1. Zygotic *rnf2* mutants lack Rnf2 protein. Lateral and dorsal view of immunostaining for Rnf2 at 2 dpf on *rnf2* mutants and wildtype siblings. Brown (DAB) staining is visible anteriorly, dorsally, and in intestinal tissue (arrow) in wildtype embryos and is absent in *rnf2* mutants. Scale bar is 1 mm.

Supplementary Figure S2. Quality check of the RNA-sequencing results and transcript levels of organ markers. (2A) Hierarchical clustering based on the Euclidian Distances of 8 replicates of wildtype embryos at 3 dpf and 7 replicates of *rnf2* mutant embryos at 3 dpf. **(2B)** Boxplots comparing the normalized counts as detected by RNA-sequencing for *fabp2, fabp10, try,* and *myl7* in wildtype and *rnf2* mutant embryos at 3 dpf.

Supplementary Figure S3. Sample filtering and quality assessment of replicates of SingleHeartsRNA-seq. (3A) Original agarose gel image of genotyping results shown in Figure 5. M indicates the DNA ladder. Samples 1-8 show representative genotyping results to identify wildtype, heterozygotes, and mutants. **(3B)** The table indicates the number of replicates that were isolated and included in generation of the sequencing library. **(3C)** The total mRNA of each sample was log transformed and samples that differed more than 2 standard deviations from the mean at the given time point were excluded from downstream analyses. The green open dots indicate the samples that were included in the analysis; the red dots indicate the samples that were removed. **(3D)** The table indicates the number of replicates that were used for downstream analyses after

filtering. **(3E,F,G)** Hierarchical clustering (Euclidian distance) of the samples used for SingleHeartsRNA-seq analyses per developmental time point.

Supplementary Figure S4. Gene expression of *tbx2a*, *tbx2b*, *tbx3a*, and *tbx5a* at 1, 2, and 3 dpf. Normalized counts as found by SingleHeartsRNA-seq for *tbx2a, tbx2b, tbx3a,* and *tbx5a* at 1, 2, and 3 dpf for *rnf2* mutant embryos and wildtype siblings (**4A,B,C**). No counts for *tbx3a* and *tbx5a* were detected at 1 dpf.

Supplementary Figure S5. Cardiac marker gene expression at 1 and 2 dpf. Normalized counts as found by SingleHeartsRNA-seq for *nppa, myl7, myh6,* and *vmhc* at 1 dpf (**4A**) and 2 dpf (**4B**) with their accompanying whole mount *in situ* hybridizations for *rnf2* mutant embryos and wildtype siblings. Scale bar is 200 µm.

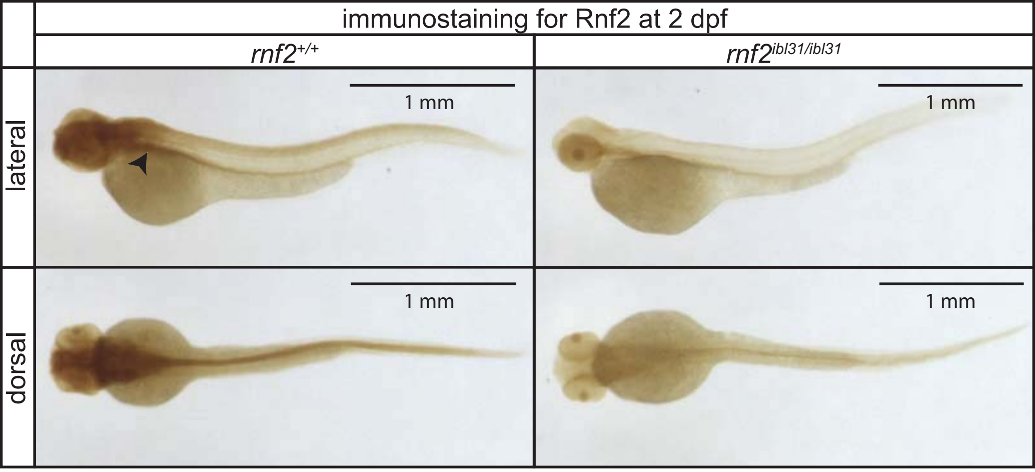

Chrispijn et al. - Supplementary Figure S1

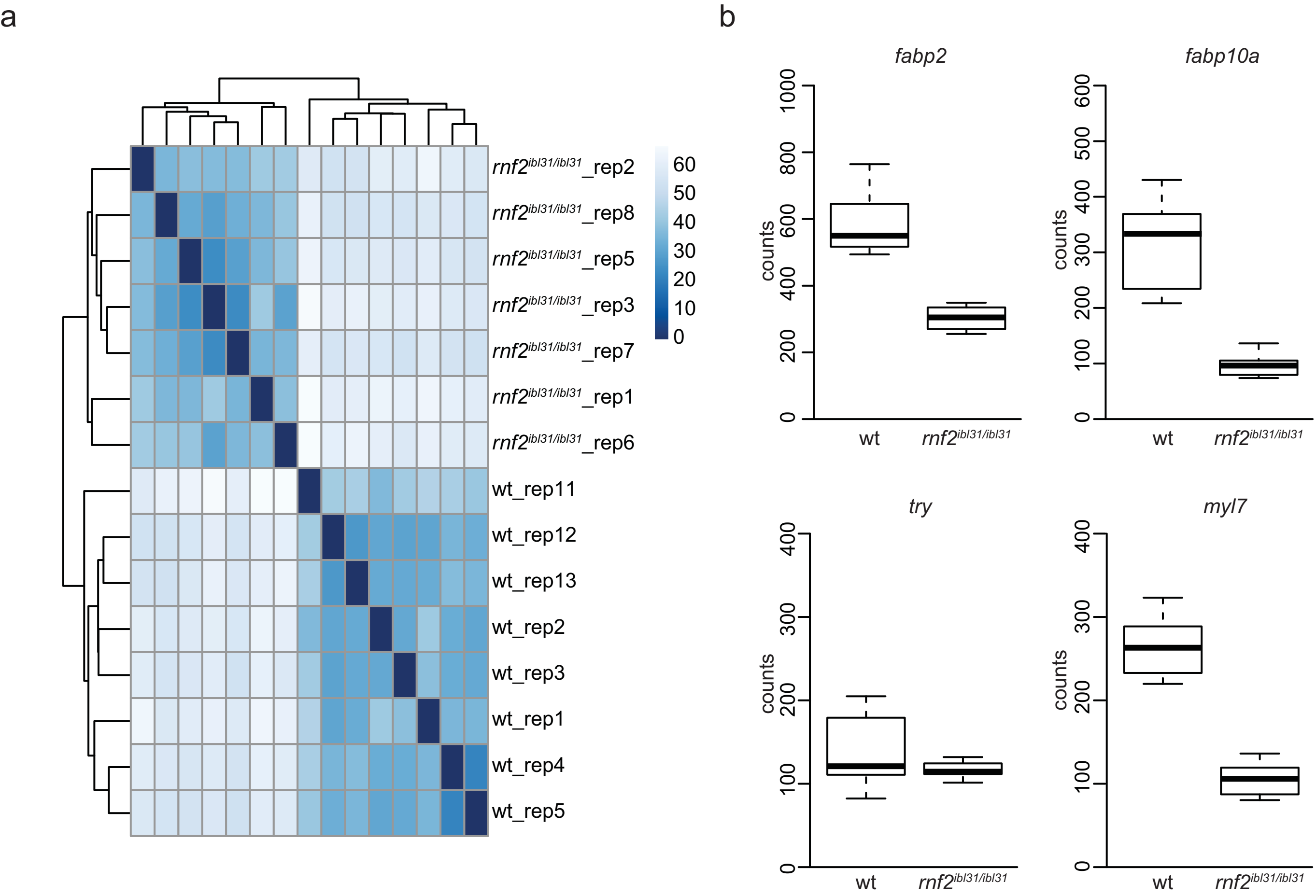

Chrispijn et al. - Supplementary Figure S2

a

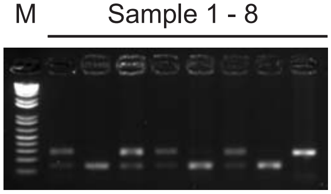

b

Number of hearts per genotype/per stage isolated		
	$rnf2^{+/+}$	$rnf2^{ibl31/ibl31}$
1 dpf	11	8
2 dpf	14	10
3 dpf	11	9

c

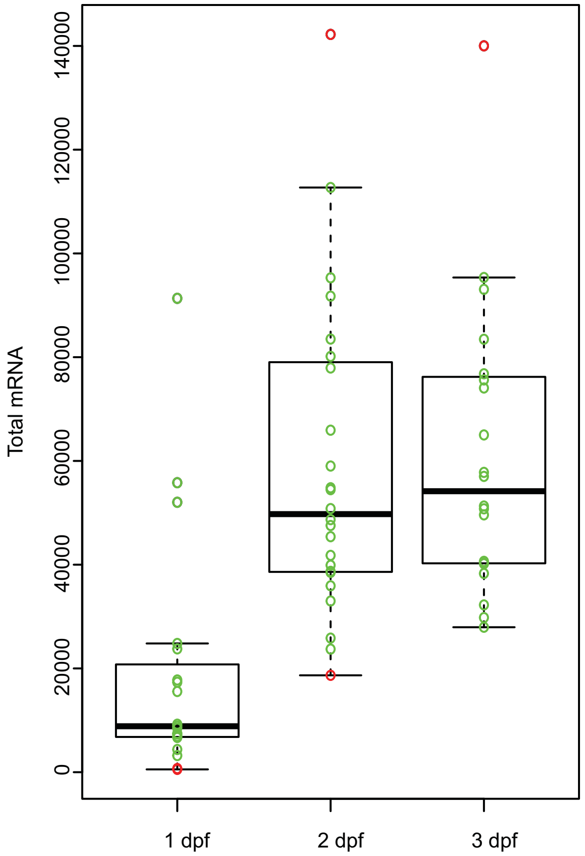

d

Number of hearts per genotype/per stage analyzed		
	$rnf2^{+/+}$	$rnf2^{ibl31/ibl31}$
1 dpf	9	8
2 dpf	13	9
3 dpf	10	9

e

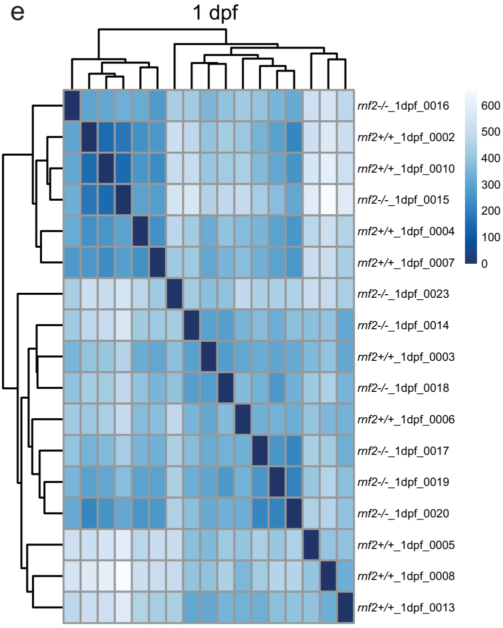

f

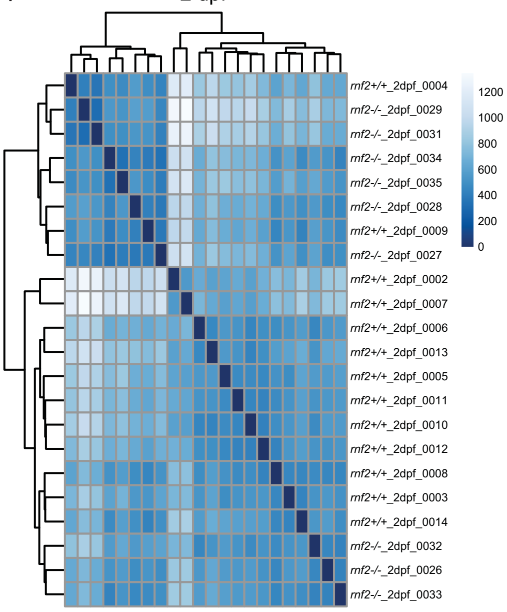

g

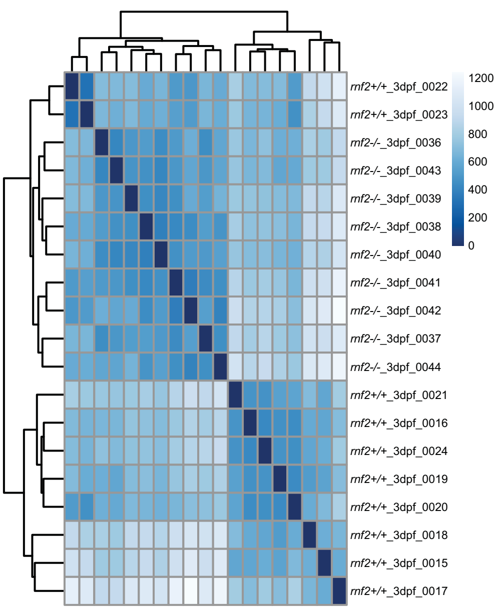

Chrispijn et al. - Supplementary Figure S3

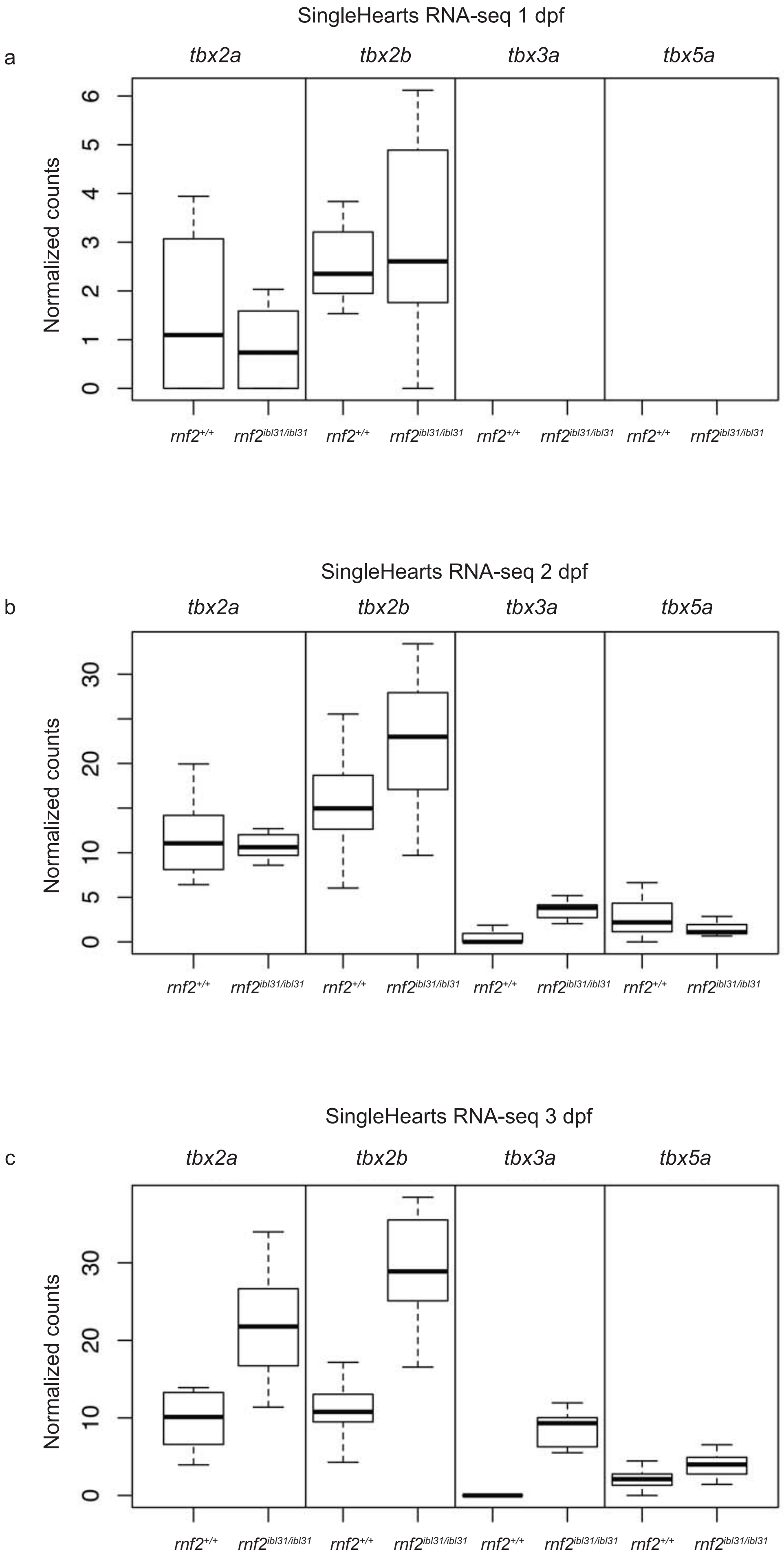

Chrispijn et al. - Supplementary Figure S5

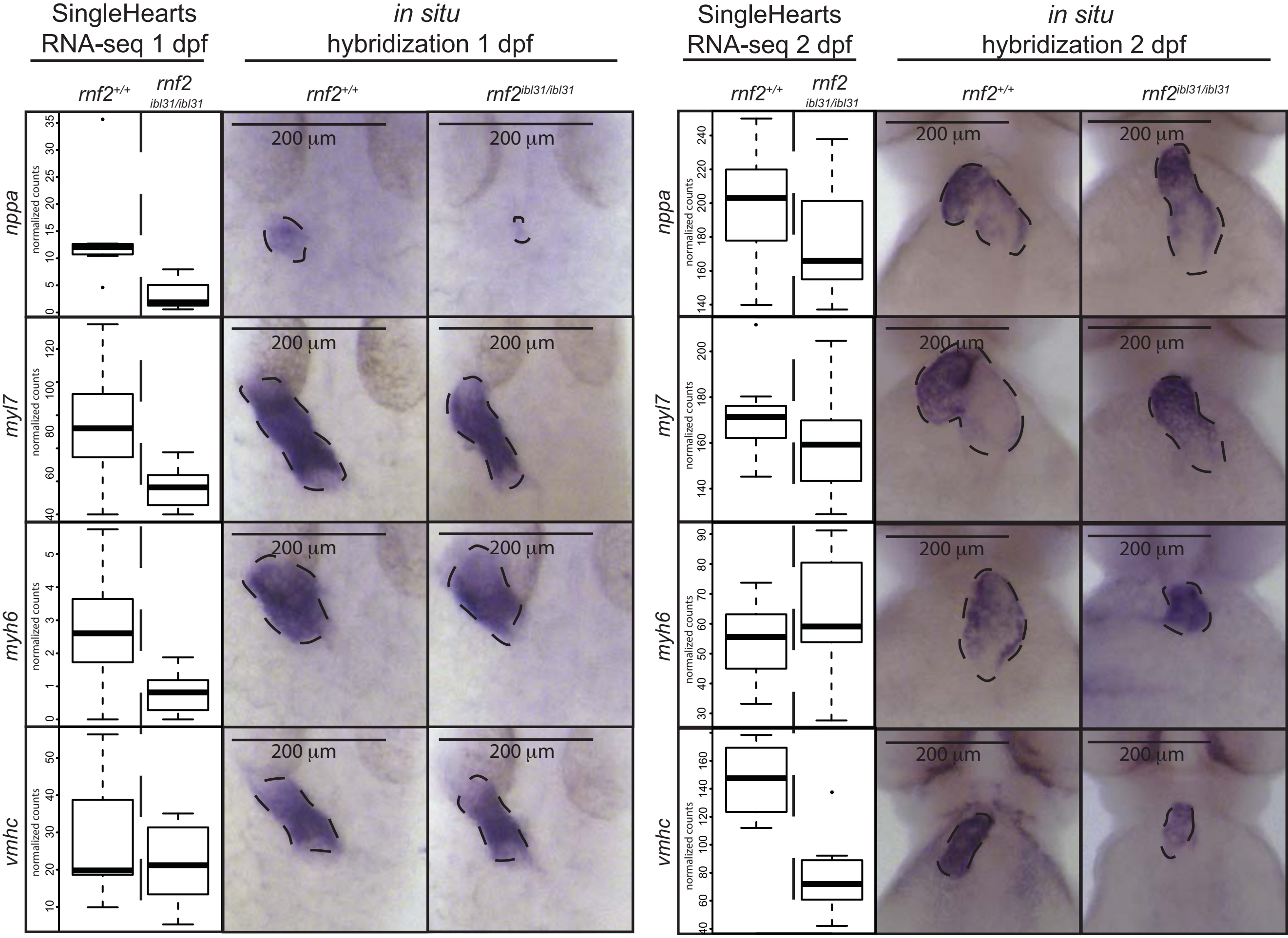

Chrispijn et al. - Supplementary Figure S4
